# Clinic-Based Retrospective Analysis of Psychopharmacology for Behavior in Fragile X Syndrome

**DOI:** 10.1155/2012/843016

**Published:** 2012-07-30

**Authors:** Elizabeth Berry-Kravis, Allison Sumis, Crystal Hervey, Shaguna Mathur

**Affiliations:** ^1^Department of Pediatrics, RUSH University Medical Center, Chicago, IL 60612, USA; ^2^Departments of Neurology and Biochemistry, RUSH University Medical Center, Chicago, IL 60612, USA

## Abstract

Fragile X syndrome (FXS) is associated with behavior that limits functioning, including distractibility, hyperactivity, impulsivity, hyperarousal, anxiety, mood dysregulation, and aggression. Medication response and side effect data were reviewed retrospectively for 257 patients (age 14 ± 11 years, range 4–60 years, 203 M, 54 F) attending an FXS clinic. Treatment success rates were defined as the percentage of positive response in the form of documented clinical report of improvement in the behavior(s) being targeted over at least a 6-month period on the medication, without side effects requiring medication discontinuance, while failures were defined as discontinuance of medication due to lack of clinical effectiveness or side effects. Success rate for treatment of targeted behaviors with trials of individual medications was 55% for stimulants, 53% for antidepressants, 62% for alpha2-agonists, and 54% for antipsychotics. With sequential trials of different medications in the same class, success rate improved to 73–77%. Side effect-related failures were highest for antipsychotics. Systematic psychopharmacologic intervention targeted to behavioral symptoms appears helpful in the majority of patients with FXS.

## 1. Introduction

Fragile X syndrome (FXS) is the most common inherited form of intellectual disability, with a frequency of about 1/4000 [1]. FXS is a single gene disorder in which a triplet repeat (CGG) expansion mutation [2] inactivates the FMR1 (fragile X mental retardation 1) gene, resulting in loss or significant reduction of expression of the FMR1 gene product, FMRP (fragile X mental retardation protein) [3, 4]. Individuals with FXS often display associated physical features such as large ears, long face, macrocephaly, and macroorchidism [5, 6]. Certain medical problems appear to be more common in FXS [5] than in normally developing populations, including strabismus, presbyopia, frequent otitis media, mitral valve prolapse, GI disturbances, and seizures. Despite wide variation in level of functioning, individuals with FXS display a fairly stereotyped cognitive profile and behavioral and personality features that are characterized by hyperactivity, anxiety, tactile defensiveness, gaze avoidance, and socialization difficulties [5–8].

Behavioral problems are often substantial in FXS, out of proportion to level of cognitive impairment [9]. Behavioral difficulties in FXS are multifactorial but can be grouped into commonly seen symptom clusters to aid treatment decisions, including (1) ADHD- (attention-deficit-and-hyperactivity-disorder) like symptoms of hyperactivity, distractibility, and impulsivity; (2) anxiety-related symptoms including sensory oversensitivity and OCD- (obsessive-compulsive-disorder) like and perseverative behaviors; (3) emotional lability with intermittent outbursts; (4) aggressive and self-aggressive behaviors. Currently, treatment strategies for individuals with FXS are solely supportive and are used to improve functioning. Although treatments designed to act specifically on the underlying neuronal defect due to FMRP deficiency are in development, there are no such treatments yet proven definitively to improve functioning in FXS. Since behavior in FXS can significantly impact functionality, symptom-based treatment to target the most problematic behavioral categories for a given individual with FXS is commonly employed in clinical practice.

Although medication management for behavior in FXS is thought to affect improvement in the clinical setting, there is a paucity of information available regarding the effects of psychopharmacology in populations with FXS, with which to guide treatment. A double-blind placebo-controlled crossover study [10] of 15 boys with FXS showed methylphenidate (Ritalin) dosed at 0.3 mg/kg to be effective in two-thirds of boys with FXS for attention and behavior, during a short one-week treatment period. The most problematic side effect was worsening of irritable behaviors. Several additional small retrospective survey studies have described a clinical response to clonidine, an alpha2-agonist (mean dose 0.15 mg), with the most significant side effect being sedation [11], and responses to fluoxetine in both males and females with FXS, with the most significant side effects being weight changes (up or down) and nausea [12]. Small open-label trials of both lithium and minocycline, drugs currently available by prescription, initiated as exploratory proof-of-concept studies based on beneficial effects of these agents in the mouse model of FXS, have also demonstrated positive effects on behavior in humans with FXS [13, 14]. Lithium, dosed to produce blood levels between 0.7 and 0.12 in 15 males with FXS, resulted in significant improvement in the total Aberrant Behavior Checklist-Community Edition (ABC-C) score, Clinical Global Impression (CGI) score; and Vineland Adaptive Behavior Scale (VABS) [13]. Similarly, treatment of 18 males and 2 females (mean age 18 ± 5, range 13–32) with minocycline demonstrated significant improvement in behavioral symptoms on the ABC-C and CGI [14]. Other early phase treatment trials of medications including mGluR5 negative modulators fenobam and AFQ056 [15, 16] and GABA-B agonist arbaclofen [17] targeted to the underlying disorder in FXS, based on work in animal models, have shown promise but are not yet available for clinical use.

Surveys of a large clinic population with FXS in Colorado [18] and an FXS clinic in Chicago [19, 20] have suggested that treatment of individuals with FXS with multiple medications for different behaviors is common. In particular, alpha2-agonists, antidepressants, stimulants, and antipsychotics are heavily used alone or in combination to produce improvement in behavioral functioning in a majority of individuals with FXS treated. In the current study, information has been collected from an expanded FXS clinic cohort to help guide clinical practice by providing additional details regarding response to these four classes of psychotropic medications. Specific goals of this analysis were to determine clinically based response rates to the four classes of psychopharmacologic agents stratified by age and sex, to evaluate frequency and types of limiting side effects for these classes of medications, and to compare response rates to specific agents within the medication classes.

## 2. Patients and Methods

Chart review was conducted regularly for all patients with FXS seen at the RUSH University Medical Center Fragile X Clinic in the years from 1991 through 2005. The patient cohort comprised the full range of functional level for FXS, ranging from nonverbal individuals with FXS to individuals (predominantly female) with IQ in the normal range. Patients were referred to the fragile X clinic for a variety of reasons including discussion of clinical expectation for patients newly diagnosed, families desiring follow up in a fragile X specialty setting, assistance with educational or vocational recommendations, genetic issues, and seizure management; however, the most frequent reason for referral and ongoing follow-up was management of behavioral problems. Behavioral management recommended was typically a combination of approaches, including psychotherapy, structured behavioral training, and optimization of the environment and curriculum. When these interventions were only partially successful or unsuccessful and significant dysfunction persisted, medication was also implemented. An FXS psychopharmacology database was utilized as a tool for clinical management to allow easy tracking of patient responses to different medications. Medications used for treatment of behavior, age at treatment, response of targeted symptoms to each medication used, and reason for failure of unsuccessful medications were entered into an FXS psychopharmacology database on a continual basis. For this study, information in the database was analyzed to generate cohort data on medication usage, response rates, and side effects in the FXS cohort. The database was locked for analysis at the end of 2005 since many of the patients followed up at the clinic began to enter clinical trials of targeted treatments, requiring changes in data collection and making it more difficult to analyze responses in a “pure” clinic setting.

Each medication was chosen for use based on clinical identification of a target problem behavior producing significant dysfunction in the individual with FXS. Stimulants were targeted to symptoms of distractibility, hyperactivity, and impulsivity; alpha2-agonists were targeted to hyperactivity, impulsivity, mild aggression, and hyperarousal and hypersensory behaviors; SSRIs (selective serotonin reuptake inhibitors) and other antidepressants were targeted to anxiety, perseverative and OCD-like behaviors, and mood lability; antipsychotics targeted outbursts, aggression, severe irritability, and other more severe aberrant behaviors. Response rates in this cohort were determined for all medications tried and grouped by gender and age for males (adults age 18 or more, and boys < 18 years), based on the age at which the medication was used. As all of the patients in the cohort were managed by only one physician, a consistent approach to medication choice, initial dose, dose titration, and assessment was utilized throughout the cohort. Follow-up regarding medication effects was obtained by phone and email communications 2–4 weeks after initiating medication and then 2 weeks after any further dose adjustments. Patients were seen for follow-up and medication review every 6–12 months (depending on distance). Response for each medication in each patient was determined as in clinical practice, with systematic questioning of parents and reports from teachers and therapists or, for older individuals, group home coordinators or job/workshop supervisors, regarding amount of improvement in targeted behaviors. Assessment was based on individualized descriptive and semi-quantitative (e.g., number of outbursts per day at school) feedback regarding the target symptom(s) from at least two sources that typically included the parents or caregivers and personnel from a school or vocational program. These reports were reviewed by the physician combined with data from interactions at clinic visits to give an impression of whether the patient was improved, unchanged, worse, or had side effects. Doses were adjusted based on this feedback. Medications were not continued if they did not produce benefit for the target symptom(s) at maximally tolerated doses. When patients were treated with multiple medications, these were always added or weaned sequentially so as to be able to ascertain the effect of each individual medication. If patients had already been on medications at the initial visit to the FXS clinic, data on medication response history was used only if specific information regarding the effects of the medication could be obtained. A positive response to each medication used was defined as documented clinical report of improvement in the behavior(s) being targeted in 2 settings, improvement sustained over at least a 6-month period on the medication, and no major side effect requiring discontinuance of the medication. Although determination of response relied ultimately on the clinical judgment of a single physician reviewing all information, this is not different from currently accepted standard of care in clinical practice.

Classes of psychopharmacologic medications analyzed included the following specific agents: stimulants included all (both short-acting and sustained release) methylphenidate preparations, mixed amphetamine salts preparations (Adderall), and dextroamphetamine preparations. Alpha2-agonists included clonidine and guanfacine (Tenex). Selective serotonin reuptake inhibitors (SSRIs) included fluoxetine (Prozac), sertraline (Zoloft), fluvoxamine (Luvox), paroxetine (Paxil), citalopram (Celexa), and escitalopram (Lexapro). Other antidepressants included tricyclics (imipramine and amitriptyline), venlafaxine (Effexor), bupropion (Wellbutrin), trazodone (Desyrel), and nefazodone (Serzone). Trazodone and nefazodone were used for behavior or for a combination of behavior and sleep problems. Antipsychotics included risperidone (Risperdal), olanzapine (Zyprexa), quetiapine (Seroquel), ziprasidone (Geodon), and aripiprazole (Abilify). Although there were a handful of individuals treated with agents not covered by this classification system, including buspirone (Buspar), anticonvulsants for mood cycling, and lithium, the number of individuals treated with these agents was too small for analysis. Furthermore, the vast majority of individuals on anticonvulsants were taking these for seizure control and behavioral responses were not being monitored.

## 3. Results

### 3.1. Description of Psychopharmacologic Treatment in the FXS Cohort

**Figure 1:**
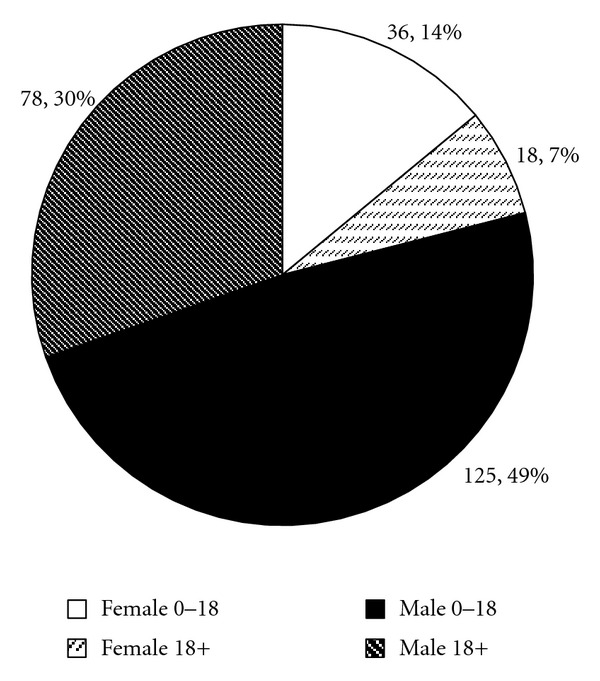
Age and gender demographics of the FXS cohort in this study.

Data from 257 total individuals in this FXS cohort was available for analysis, including 203 males and 54 females ranging from one to sixty years of age.  Figure 1 shows the composition of the cohort, fractionated into gender and age groups. Of the subjects in the cohort, 52.9% (57.6% of males and 35.2% of females) were treated with a stimulant, 22.6% (26.1% of males and 9.3% of females) were treated with an antipsychotic, 47.9% (49.3% of males and 42.6% of females) were treated with an antidepressant, and 20.2% (25.1% of males and 1.9% of females) were treated with an alpha2-agonist. In all, 72% (75% of males and 59% of females) had been treated with at least one psychopharmacologic agent for behavioral management at some time. In total, 208 trials of stimulants in 145 patients (5 adult males, 119 males < 18 and 21 females), 230 trials of antidepressants in 133 patients (26 adult males, 82 males < 18 and 25 females), 100 trials of antipsychotics in 64 patients (19 adult males, 40 males < 18 and 5 females), and 52 trials of alpha2-agonists in 52 patients (1 adult male, 50 males < 18 and 1 female) were conducted through the clinic. Males under 18 most commonly had treatment trials of stimulants. Males over 18 and females most commonly had treatment trials of antidepressants.

### 3.2. Response to General Classes of Psychopharmacologic Medications

**Figure 2:**
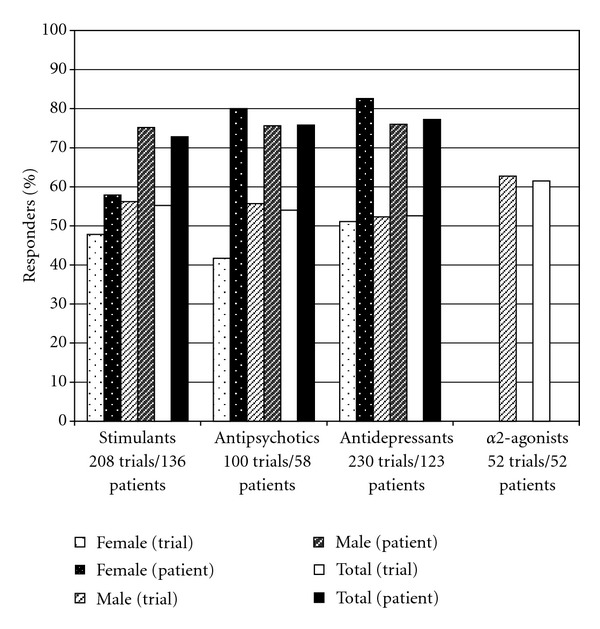
Response rate for major classes of psychopharmacological agents utilized. Response rate for “trials” indicates percent of positive responses for individual trials of medications within the class. Response rate for “patients” indicates percent of patients who responded positively to any medication within the class. Only the trial response rate is shown for alpha2-agonists because virtually all of these treatments were with clonidine and there were no patients with trials of two different medications in this class, thus the patient response rate is the same as the trial response rate for this medication class. No response data is shown for alpha2-agonists in females because no females were treated; the fraction of responders is zero for alpha2-agonists in males > 18.

For stimulants, antidepressants, and antipsychotics, there are lower response rates for both males and females when response is analyzed based on success of individual trials of medication in the drug class than when response is analyzed based on successful treatment of patients with any medication within the drug class (Figure 2). No individual had more than one trial of an alpha2-agonist and thus success rate of medication trials is the same as the rate of successful patient treatment for this drug class. Overall 99/136 (73%) patients with FXS responded to at least one stimulant 95/123 (77%) to an antidepressant, 44/58 (76%) to an antipsychotic, and 32/52 (62%) to an alpha2-agonist. Females with FXS showed lower response rates than males to stimulants although females and males showed similar response rates to antidepressants and antipsychotics (Figure 2).

**Figure 3:**
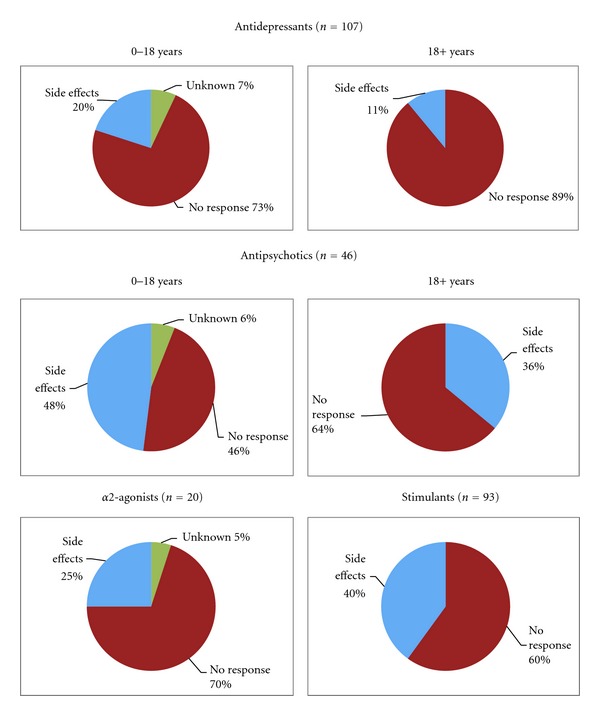
Reason for failure of various classes of medications during treatment trials. Green areas of charts indicate percent of individuals for whom no follow-up information was available, Red areas indicate failure due to medication ineffectiveness, and Blue sections of charts represent individuals failing due to a side effect that limited treatment. For antidepressants and antipsychotics data is presented separately for children and adolescents less than 18 and adults 18 and over to compare rates of intolerable side effects in these age groups. For alpha2-agonists and stimulants, only 1 and 5 individuals, respectively, aged 18 or over failed treatment, all due to medication ineffectiveness. Therefore, age groups were not presented separately for these medication classes.

In total, 93/208 stimulant trials (45%) failed, 107/230 antidepressant trials (47%) failed, 20 alpha2-agonist trials (38%) failed, and 46/100 antipsychotic trials (46%) failed. The majority of failures for all medication classes were because the treatment was not helpful (Figure 3). There was a higher frequency of side-effect-related failures for antipsychotics and stimulants and very low frequencies of side-effect-related failures for alpha2-agonists and antidepressants (Figure 3). Side effects were more frequently the reason for medication failure for individuals less than 18 years than for adults over 18 years (Figure 3).

Side effects most commonly observed with stimulants included appetite suppression with occasional weight loss, stomach discomfort, lethargy, suppression of exuberance, reduced speech output, and aggravation of anxiety, perseverative, irritable, and aggressive behaviors. Stimulants were virtually never discontinued because of appetite problems, and in fact, in many cases, this was viewed as a beneficial side effect. The most common side effect resulting in stimulant discontinuance for individuals with FXS was aggravation of anxiety/perseveration/irritability. The most common side effects observed with antidepressants were nausea, diarrhea, sedation, and aggravation of impulsive and disinhibited behavior. For the most part, gastrointestinal side effects and lethargy were mild and often manageable with dose or timing modifications. The side effect that resulted in the most antidepressant discontinuances in the FXS cohort, particularly for SSRIs but also observed for other antidepressants, was severe disinhibited behavior. Aggravation of sleep problems was not commonly observed in this FXS cohort with either stimulants or antidepressants. The most common side effects observed for antipsychotics were nausea, vomiting, lethargy, and weight gain. The most common side effect resulting in antipsychotic discontinuance for individuals with FXS was problematic weight gain. Four instances of extrapyramidal side effects were observed with antipsychotic treatment, and these involved parkinsonian symptoms including bradykinesia, mild rigidity, problems with initiation of movement including swallowing, and aggravation of baseline coordination deficits. These effects occurred at very high doses of olanzapine (one patient) and aripiprazole (one patient) but were seen at doses as low as 0.5 mg of risperidone for the other two patients. Resting tremor was also observed in relation to high-dose aripiprazole treatment in the patient described above, despite excellent behavioral response. Tardive dyskinesia was never observed in any individual with FXS, even with antipsychotic treatment for as long as 10 years. The only side effect requiring discontinuance of an alpha2-agonist in this FXS cohort was persistent sedation that did not abate over the first weeks of treatment.

### 3.3. Response to Stimulants

Data on treatment and response to different types of stimulants was analyzed in an attempt to determine whether there was an advantage to treatment with either MPH (methylphenidate) preparations (MPH, includes generics, Ritalin, Ritalin SR, Ritalin LA, Metadate, Metadate CD, Methylin, Concerta, Focalin, and Focalin XR) or APH (amphetamine) preparations (APH, includes Adderall, Adderall XR, Dextrostat, and Dexedrine) in the FXS population. Overall a larger percentage of individuals, both males and females, were treated with MPH preparations (2.6% adult males, 76.8% males <18, 29.6% females) than with APH (3.5% adult males, 60.8% males <18, 16.7% females), although a significant percentage of individuals had trials of both types of stimulant. There were no differences between patterns of use of MPH versus APH in males and females with FXS.

**Figure 4:**
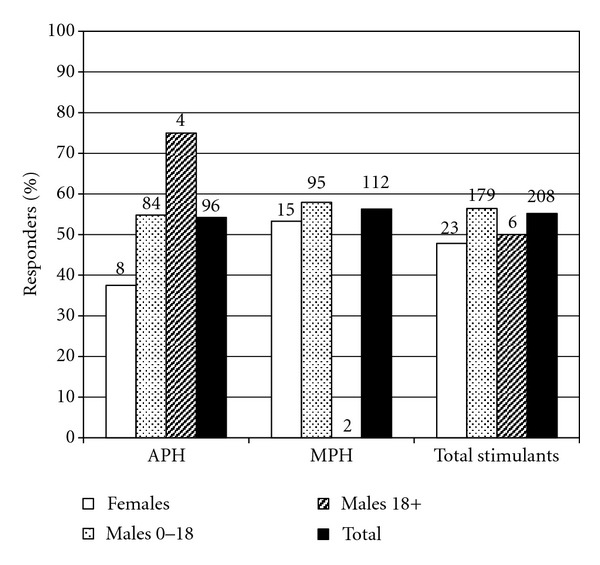
Response rates in patients with FXS to trials of different types of stimulants, fractionated by age and gender. Numbers above bars represent total number of patients in the indicated category treated with the stimulant type.

In total, 63 of 112 trials of MPH (56%) and 52 of 96 trials of APH (54%) were successful (Figure 4), as compared to the 73% (Figure 4) of individuals tried on either type of stimulant who were successful on at least one. Response rates to MPH and APH were similar in boys with FXS, although adults, both male and female, showed a better response rate to APH (Figure 4).

Reasons for failing treatment trials with MPH or APH showed nearly identical patterns. The majority (61% for APH, 60% for MPH) of failures for both stimulant types were because the treatment was not helpful (no change from baseline ADHD-like behavior), while the remainder failed treatment primarily because of side effects (including worsening of irritability and hyperactivity).

**Figure 5:**
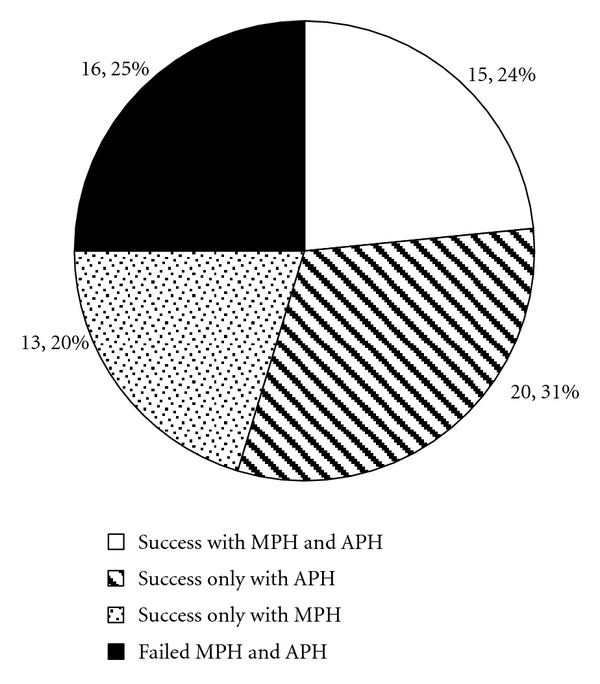
Results of sequential stimulant trials for the subgroup of individuals with FXS who had trials of both APH and MPH preparations (*N* = 64).

The outcome of independent trials of MPH and APH was assessed for those individuals with FXS who had trials of both stimulant types at different times (Figure 5). Starting doses of MPH were usually about 0.2 mg/kg/dose for short-acting preparations and 0.4 mg/kg/dose for sustained release preparations with titration up to as much as 0.5 mg/kg/dose or 1 mg/kg/dose, respectively. For APH starting doses were about typically 0.125 mg/kg/dose for short-acting preparations and 0.25 mg/kg/dose for sustained release preparations with titration up to as much as 0.4 mg/kg/dose or 0.8 mg/kg/dose, respectively. While some individuals with FXS were simply nonresponsive or unable to tolerate stimulants and some did well on either stimulant type, there were distinct subgroups of individuals with FXS who were successful on one stimulant class but not the other. Of patients with FXS who were tried on both stimulant types, 52% were successfully treated only with one type (32% APH and 20% MPH, Figure 5). Profiles of side effects observed were similar for APH and MPH.

It should be noted that 6 trials of atomoxetine with starting dose typically about 0.5 mg/kg/day were carried out in this FXS cohort (data not shown, 4 males, 2 females) and all of these were unsuccessful, with the majority failing due to aggravation of irritable, moody, and aggressive behaviors.

### 3.4. Response to Antidepressants

**Figure 6:**
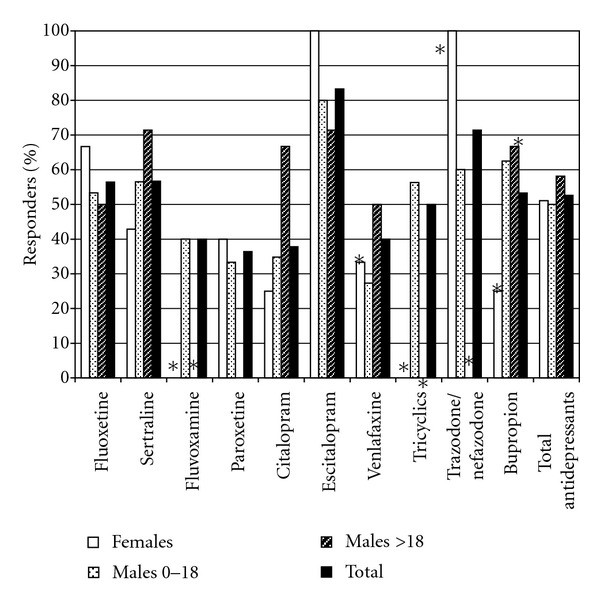
Response rates to different antidepressants, fractionated by age and gender. For bars with an asterisk, there were <5 trials of the indicated medication for the patient group represented by the bar.

Fluoxetine, sertraline, and citalopram were the most commonly used antidepressants (Figure 6). Low apparent response rates for paroxetine and fluvoxamine and high apparent response rates observed for escitalopram and trazodone/nefazodone may have to do with the relatively low number of trials conducted with these agents. Otherwise, response rates were fairly similar for different agents within the antidepressant class, and similar for SSRI and non-SSRI antidepressants. Although sertraline and fluoxetine had relatively similar response rates across the total cohort, females had higher response rates to fluoxetine while males had higher response rates to sertraline. This finding corresponds to differences in phenotype between females, who tend to be shy and withdrawn and may benefit from activating effects of fluoxetine, and males, who tend to be hyperactive and impulsive and may require a less activating SSRI. There was no consistent difference in types of side effects observed with different antidepressants, although some individuals who became excessively disinhibited on SSRIs were able to be successfully treated with venlafaxine, a tricyclic antidepressant, trazodone, nefazodone, or bupropion.

### 3.5. Response to Antipsychotics

**Figure 7:**
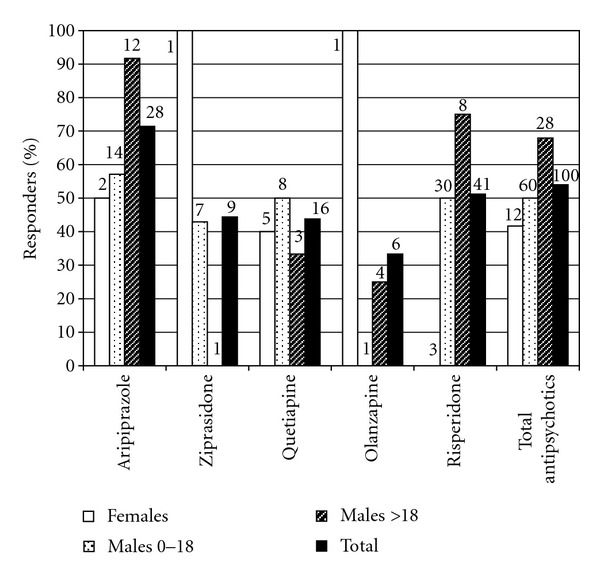
Response rates to different antipsychotics, fractionated by age and gender. Numbers above bars represent total number of patients in the indicated category treated with the antipsychotic agent.

Multiple atypical antipsychotics were used with risperidone and aripiprazole being most frequently used (Figure 7). Aripiprazole had the highest overall response rate. Olanzapine had a somewhat lower rate of overall successful trials in individuals with FXS, because it was often discontinued due to excessive weight gain. Risperidone was particularly helpful for boys < 18 years with FXS for irritability, perseveration and aggression, and autistic features. Apparent very high response rates in many of the female subgroups are clearly artificially elevated due to very low numbers of trials in these groups. Insufficient females were treated with each individual agent in the antipsychotic class to draw any conclusions about response rates to individual medications. The side effect of weight gain was much more commonly seen with olanzapine and risperidone than quetiapine, ziprasidone, and aripiprazole.

**Figure 8:**
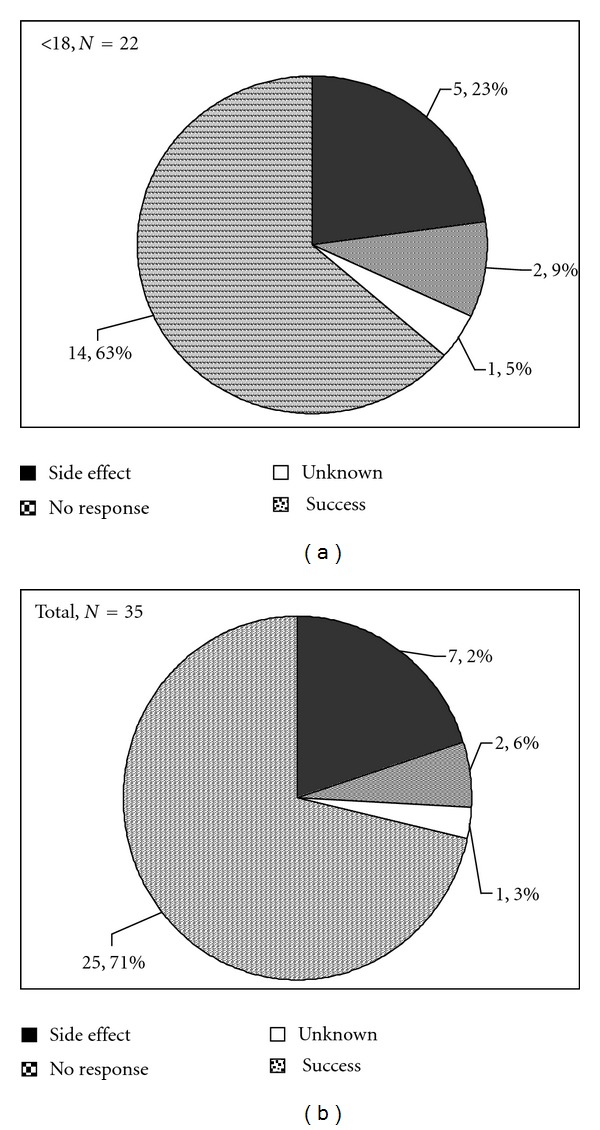
Response and failure rates in patients with FXS treated with aripiprazole, fractionated by age. This figure includes response data for aripiprazole from 7 additional patients with FXS started on this treatment between the end of December of 2005 and July of 2006.

Because aripiprazole is a reasonably new agent and data were limited in the 1999–2005 cohort, responses to aripiprazole were analyzed in additional patients with FXS receiving this treatment through July of 2006. High response rates were confirmed in the larger series (Figure 8); however, about 20% of patients with FXS failed treatment with aripiprazole, predominantly due to aggravation of aggressive, perseverative, and irritable behavior. The high response rate observed in our study is consistent with results of an open-label trial of aripiprazole in 15 patients with FXS and no concomitant medication use, in which substantial improvement in irritable, hyperactive, and other behaviors was noted on the ABC-C, CGI, and several other scales [21].

## 4. Discussion

This paper presents a retrospective analysis of clinically assessed results of psychopharmacological treatment for a large cohort of males and females with fragile X syndrome (FXS). From the data presented here we can conclude that males with FXS under the age of 18 years most commonly exhibit ADHD-like symptoms that are treated with stimulants and males over 18 years and females with FXS most commonly exhibit mood and anxiety symptoms dictating treatment with antidepressants. This is consistent with the patterns of medication usage observed in smaller FXS cohorts presented previously [18–20].

Successful treatment of targeted behaviors in FXS ranged from 53 to 62% for trials of different classes of psychoactive medications. The rate of success, however, improved with sequential trials of medications within a class such that ultimately targeted behaviors were perceived as improved for about 73–77% of individuals with FXS. These response rates to the studied medication classes are similar to those presented in a smaller FXS cohort previously [20]. This would suggest that psychopharmacology targeted to ADHD-like and anxiety/mood symptoms in FXS can be quite helpful and appears to have fairly high efficacy in a clinical setting although several trials of medicines from a particular class may be needed to achieve successful treatment without problematic side effects. The majority of treatment trials fail because the medication is not helpful for the targeted symptom although over a third of stimulant and antipsychotic trials fail because of side effects. Side effects are a more frequent reason for medication discontinuance in children and adolescents than in adults, consistent with prior reports of similar effects [22].

Stimulants were helpful in a clinical setting to target distractibility, hyperactivity, and impulsive behavior in this study as 73% of individuals with FXS responded without major side effects to some preparation of stimulant. This is similar to the 67% response rate seen with methylphenidate in the one controlled study [10]. Although adult males with FXS tend to be less overactive and more anxious leading to a medication shift towards the use of antidepressants, some individuals are helped substantially by the use of stimulants, even into their thirties or forties. In a recent report [23], boys with FXS on stimulants had better attention, lower motor activity levels, and higher academic test scores on medicated days versus unmedicated days, although levels of physiological arousal were unaffected by stimulant treatment. Electrodermal studies measuring an enhanced sweat response to stimuli in children with FXS did show a decrease in response toward normal after stimulant treatment [24]. Taken together, currently available information suggests that stimulants are quite helpful in managing distractibility and hyperactivity symptoms in a subgroup of boys and girls with FXS presenting with prominent difficulty in these behavioral domains.

In some individuals with FXS, stimulants exacerbate anxiety, irritability, or aggressive tendencies and must be abandoned. Indeed in this study 40% of failed stimulant trials occurred because of side effects, which mostly consisted of aggravation of anxiety/irritability/aggressiveness. Stimulants now come in many different long-acting forms that may be quite useful in eliminating swings in mood and behavior during the day seen on multiple-dose regimens of fast-acting preparations. Stimulants are thought to induce excessive side effects or may not be effective in children with FXS less than five or six years old, although they may be quite effective if reintroduced at an older age. The number of patients less than 5 years of age treated in our series is too low to clarify this issue (data not shown). In populations with nonspecific mental retardation, stimulants have been shown to be more effective in individuals with higher IQ, while side effects are more problematic in those with lower IQ [25, 26]. Since there is a wide range of functioning in FXS and outpatient clinic populations are likely biased toward higher functioning individuals (lower functioning individuals may be institutionalized or home-bound), stimulant response rates presented here accurately reflect a clinic population but may be somewhat high for the general population of males with FXS.

Data from this study show that MPH and APH preparations have about the same overall response rates and the same profile of reasons for failure, although adults with FXS may be more likely to respond to APH. It also appears that distinct groups of patients with FXS respond to or have side effects on only one of APH or MPH, suggesting that sequential trials of both will raise overall response rate and are indicated before abandoning treatment. Further, if an adolescent or adult with prominent attention problems is no longer responding well to an MPH preparation, it would be reasonable to try switching to APH before using another category of medication.

Antidepressants were helpful in a clinical setting to target anxiety, compulsive and perseverative behaviors, and mood symptoms in this study as 77% of individuals with FXS responded without major side effects limiting treatment to at least one antidepressant. SSRIs were most commonly utilized and were the most common form of treatment for females and adults with FXS. SSRIs appear to be particularly helpful for social anxiety and withdrawal in females with FXS, and fluoxetine has been previously reported to be successful for selective mutism in females with FXS and extreme shyness [27]. Response rate to individual trials of antidepressants, including both SSRIs and other antidepressants, was about 53% in this study, consistent with data from a self-report survey of effects of fluoxetine in adults with FXS, which revealed improvements in anxiety and mood in about 70% of treated individuals [12]. Response rates in FXS appear to be similar to the 60–70% response rate to fluoxetine observed for subjects with autistic disorder or mental retardation in an open-label trial [28] and the 53% response rate to fluvoxamine observed in a placebo-controlled trial for adult subjects with autistic disorder [29]. A parallel study of this drug in children and adolescents with autism showed a lack of efficacy and significant adverse effects, including behavioral activation. A recent unblinded prospective study of effects of sertraline in 12 children with FXS [30] showed improvement in emotional and behavioral parameters after starting sertraline, although some had to discontinue treatment due to disinhibition with increased impulsivity. Taken together, available studies suggest that SSRIs can be useful for management of anxiety and behavioral/emotional symptoms in individuals with FXS.

The predominant side effect of SSRIs observed in this FXS cohort was activation with an increase in hyperactivity and disinhibited behaviors, which may be more pronounced with fluoxetine. Less activating SSRIs, such as sertraline or escitalopram, may be better in individuals with FXS and higher levels of hyperactivity and impulsive behavior. For individuals who are too disinhibited on SSRIs, other antidepressants may be successful treatments that do not produce the disinhibited behavior and, in this study, had similar response rates when compared to the SSRIs. Tricyclic antidepressants can also work well for bedwetting and sleep dysregulation [31] although EKGs must be monitored, as sudden death, presumed due to cardiac dysrhythmias, has been described in rare individuals with FXS. Bupropion, which increases dopamine levels more than other antidepressants, can help with both focusing and mood/anxiety issues. Several teenagers with FXS in this cohort have done quite well with bupropion to target both of these symptom areas. Bupropion can precipitate seizures in at-risk individuals and therefore should not be used in individuals with FXS and active seizures. Trazodone can help with sleep dysregulation and anxiety and has been found helpful for managing aggression in children with severe behavioral disturbance [32]. A good response rate to trazodone, particularly for nocturnal sleep disturbances, was seen in the current study for the small number of individuals with FXS treated.

Antipsychotics are generally reserved for individuals with FXS who exhibit more extreme behaviors and use rates are thus lower than for stimulants and antidepressants. Antipsychotics were helpful in a clinical setting to target irritability, aggression, and perseverative behaviors in this study, as 76% of individuals with FXS responded to at least one antipsychotic, without side effects requiring withdrawal. Risperidone was effective clinically in FXS with high response rates for aggressive behavior in older males with FXS and other aberrant and undesired behaviors in young boys with FXS and autistic traits. This is consistent with the finding that risperidone is safe and effective for aggressive and aberrant behaviors in a double-blind placebo-controlled trial in individuals with autism [33]. Other atypical antipsychotics (quetiapine, ziprasidone) have less effect on weight and were helpful for aggressive behavior in some patients with excessive weight gain on risperidone or olanzapine. Aripiprazole also has less effect on weight and appears to have rather high response rates in individuals with FXS, for whom, because of its unique pharmacological profile, it may target multiple problematic areas including distractibility, aggressive and agitated behavior, and aberrant social behaviors [6]. Some individuals with FXS, however, simply cannot tolerate aripiprazole because of side effects similar to those seen with stimulants including aggravation of irritable and perseverative behaviors. This effect is not seen with other atypical antipsychotics presumably because aripiprazole is the only atypical with partial dopamine agonist activity. Although the newer atypical antipsychotics are less sedating and have a more favorable motor side effect profile than older antipsychotics like haloperidol and thioridazine, side effects were still more frequent for atypical antipsychotics than for other classes of medications in this FXS cohort.

Alpha2-agonists, clonidine and guanfacine, showed about 62% efficacy in a clinic setting in treating hyperactive, hyperaroused, hypersensitive, impulsive, and aggressive behaviors due to overarousal in young boys with FXS, consistent with a survey study showing an 80% response rate for management of hyperarousal and hyperactivity. The results in studies of FXS are consistent with substantial improvement in hyperactivity and impulsivity seen in a double-blind placebo-controlled crossover trial of clonidine in hyperactive children with nonspecific mental retardation [34]. Alpha2-agonists may be particularly effective in young children who do not tolerate or respond to stimulants and can be quite helpful for sleep problems, although sedation can also be a problematic side effect. Sedation is often most prominent in the first few weeks after medication initiation and dose increases and often abates after that. Likewise, in some patients alpha-agonists provide only temporary benefit for sleep management.

This study is clearly limited by its retrospective nature, lack of objective valid/reliable outcome measures, and lack of tracking of dosing information. Although care was taken to try to get documentation of medication effects from at least two persons working with the individual with FXS, there is likely to be placebo effect and response rates are probably somewhat high. Also this study cohort was treated over a period of 15 years during which pharmacological treatment evolved with more options available. Thus, there may have been less opportunity to identify a successful regimen for patients for whom treatment was attempted earlier in the time period. Although this clinic population contained patients with a broad range of functional level, it is likely somewhat biased toward higher functioning patients with FXS who are able to get into clinic, are not institutionalized, and are expected to be more treatable, thus raising response rates. There are a number of medications, including anticonvulsants, buspirone, and propanolol, that have been anecdotally used for behavior in, but they were not included in this analysis as very few patients were treated with these medications for behavior in this fragile X patient cohort.

Current therapy in FXS is predominantly supportive or symptom based, and no therapy currently exists that has been shown to improve cognitive ability in FXS. As information regarding the specific neural functions of FMRP has become available, more directed pharmacological interventions have been explored, potentially acting on GABA [35] or glutamatergic receptors or signaling pathways [36, 37] implicated in mechanisms of synaptic dysfunction generated by the absence of FMRP. Hence, lithium, thought to reduce excessive group 1 mGluR-mediated translational activation and reverse abnormal phenotypes observed in the *Fmr-1* knockout mouse model [32], showed positive effects on behavior and a measure of auditory memory in an open-label pilot trial [13]. Minocycline, which inhibits excessive activity of MMP9, normalizing some synaptic and behavioral phenotypes in the *Fmr-1* knockout mouse model [38], also had positive effects on behavioral symptoms such as irritability, hyperactivity, and stereotypy in FXS in a small open-label trial [14]. Although both lithium and minocycline are currently available as prescription therapies, additional placebo-controlled trials will be necessary to definitively demonstrate effectiveness and justify general use for specific symptoms. Memantine [9] and riluzole [21] were utilized empirically in small pilot open-label studies based on effects on glutamate/GABA mechanisms and shown to have variable mild effects on some behavioral and biomarker measures that were not considered definitive enough to pursue further study. An open-label study of acamprosate, which acts on both GABAergic and glutamatergic mechanisms, in 3 patients with FXS suggested improvement in behavioral symptoms and increased linguistic function [39] prompting additional ongoing study. Early trials of mGluR5 negative modulators, which block excessive mGluR5 signaling that results from absence of FMRP and have reversed numerous synaptic and behavioral phenotypes in the *Fmr-1* knockout mouse model [36, 37], have been initiated. An open-label, single-dose trial of fenobam demonstrated an improvement in prepulse inhibition and subjective improvement in anxiety-related behaviors after the dose, but no clinically significant adverse effects [15]. Encouraging results were also obtained from an early phase double-blind placebo-controlled crossover design trial of AFQ056 in 30 patients with FXS (Novartis, a selective mGluR5 antagonist). Individuals with a fully methylated *FMR1* promoter showed improvement on the primary outcome, the ABC-C, the CGI, and numerous other behavior scales. Individuals with a partially methylated *FMR1* promoter had a more variable response [16]. Arbaclofen, a GABA-B agonist that acts presynaptically to decrease glutamate release and mGluR activation, also reverses behavioral phenotypes in the *Fmr-1* knockout mouse [40] and showed benefits in social functioning on the ABC-C and VABS in a phase II placebo-controlled crossover study involving 63 patients with FXS [17]. Based on results from these early trials, arbaclofen and several mGluR5 negative modulators are now undergoing further development in phase III trials. Until such agents are available, however, data presented here, in combination with available literature suggests that psychopharmacological medication treatment targeted to specific problem behaviors can improve these behaviors in a clinical setting in a substantial fraction of individuals with FXS and thus can be a valuable supportive intervention to maximize functioning in FXS.
